# Beyond Back Splicing, a Still Poorly Explored World: Non-Canonical Circular RNAs

**DOI:** 10.3390/genes11091111

**Published:** 2020-09-22

**Authors:** Annie Robic, Christa Kühn

**Affiliations:** 1 GenPhySE, University of Toulouse, INRAE, ENVT, 31326 Castanet Tolosan, France; 2 Institute of Genome Biology, Leibniz Institute for Farm Animal Biology (FBN), 18196 Dummerstorf, Germany; kuehn@fbn-dummerstorf.de; 3 Faculty of Agricultural and Environmental Sciences, University of Rostock, 18059 Rostock, Germany

**Keywords:** intron lariat, lariat-derived circRNA, back splicing, annotation of circRNAs, intron circle, sisRNA, sub-exonic circRNA

## Abstract

Most of the circRNAs reported to date originate from back splicing of a pre-mRNA, and these exonic circRNAs are termed canonical circRNAs. Our objective was to provide an overview of all other (non-canonical) circRNAs that do not originate from the junction of two exons and to characterize their common properties. Those generated through a failure of intron lariat debranching are the best known, even though studies on them are rare. These circRNAs retain the 2′–5′ bond derived from the intron lariat, and this feature probably explains the difficulties in obtaining efficient reverse transcription through the circular junction. Here, we provide an unprecedented overview of non-canonical circRNAs (lariat-derived intronic circRNAs, sub-exonic circRNAs, intron circles, tricRNAs), which all derive from non-coding sequences. As there are few data suggesting their involvement in cellular regulatory processes, we believe that it is early to propose a general function for circRNAs, even for lariat-derived circRNAs. We suggest that their small size and probably strong secondary structures could be major obstacles to their reliable detection. Nevertheless, we believe there are still several possible ways to advance our knowledge of this class of non-coding RNA.

## 1. Introduction

In 2012, advances in high throughput sequencing revealed the presence of circular RNAs (circRNAs) in mammalian cells [1]. During splicing of linear primary transcripts (pre-mRNA), introns (non-coding regions) are spliced out in the form of lariat intronic RNA and exons are joined together [2]. These two aspects of splicing explain the genesis of two types of circular transcripts, since circRNA is probably a natural byproduct of the splicing process in all eukaryotes [3,4]. Exonic circRNA is generated by the circularization of one or several exons through a back splicing process: the end of an exon is joined to the beginning of an upstream exon [5]. When intron lariats escape degradation due to the failure of intron debranching, they may become circRNA precursors [6].

Five seminal articles paved the way for further detailed investigations of circRNA by describing three important steps in circRNA characterization: (1) The year 2012 saw the first characterization of exonic circRNAs and back splicing [1]; (2) In 2013, the first characterization of circRNAs derived from intron lariats was published [6]; (3) In 2013, circRNAs were shown to have functional relevance ([7,8,9], as reviewed by [10,11]). Since 2012, circular RNAs have been the topic of a large number of publications, but in three reviews selected among those published in 2019, only two [12,13] mention the lariat-derived circRNA characterized [6], while the last focuses exclusively on exonic circRNAs [14]. Like the authors of these reviews, we noticed that an increasing number of studies investigated only exonic circRNAs that become canonical circRNAs. However, a literature search in PubMed yielded several hundred articles that referred to “intronic circular RNA”. We start by showing that knowledge of intron-derived circRNAs has progressed, but that it is important to avoid masking this knowledge by articles that do not truly identify this type of circRNA. Here, we define non-canonical circRNAs as circular transcripts obtained by pathways other than back splicing. The aim of this review is to highlight the existence of different types of non-canonical circRNAs and to identify their common properties.

## 2. Some circRNAs Are Derived from Intron Lariats

**Figure 1:**
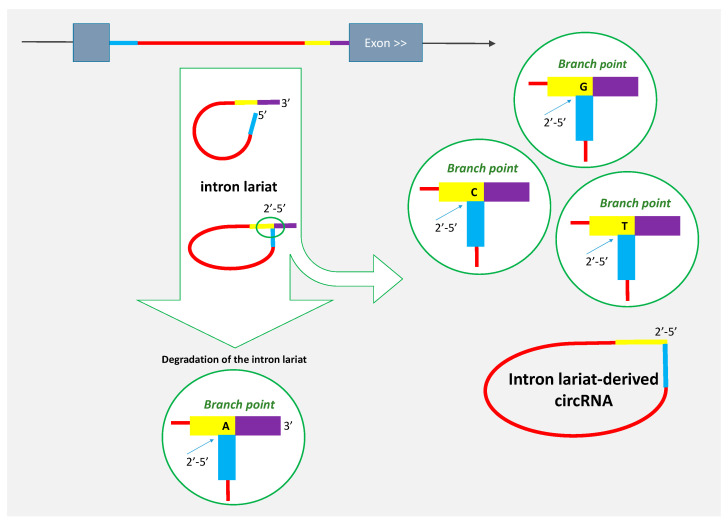
Genesis of lariat-derived circRNA. When intron lariats escape degradation through failure of intron debranching, they can become circRNA precursors. The intron excision from pre-mRNA releases a lariat molecule, in which the branch point nucleotide, predominantly an adenosine, is linked 2′–5′ to the 5′ end of the intron [15,16]. The lariat debranching enzyme is hypothesized to be incapable of hydrolyzing the 2′–5′ bond when the branch point nucleotide is not an ‘A’ [15].

In 2013, Zhang et al. [6] reported the first circular and intronic transcripts. Spliceosome-mediated intron excision from pre-mRNA releases a lariat molecule, which is a circular RNA produced by branching from the 5′ end of the intron close to the 3′ end of the intron and that keeps a 3′ tail (Figure 1) [2]. The branch point nucleotide is mainly an adenosine (Figure 1) and is always linked 2′–5′ to the 5′ end of the intron (Figure 1) [15,16]. The intron lariat is usually attacked by a debranching enzyme and by exonucleases. Thus, the lariat is only an intermediate molecule that is usually rapidly degraded, but some introns appear to be capable of evading this turnover pathway to form stable intronic sequence RNA (sisRNA) ([17], reviewed in [18,19]).

To become circular sisRNA, the lariat escapes the action of the debranching enzyme (Figure 1). Several studies showed that lariat-derived circRNAs originate from introns that do not use an adenosine at the branch point but rather a ‘C’, or more rarely, a ‘G’ or ‘T’ [6,20,21,22,23,24,25]. The lariat debranching enzyme is hypothesized to be incapable of hydrolyzing the 2′−5′ bond at these residues [15]. Intron lariats that are able to escape debranching appear to include a signature of a 7-nt GU-rich motif near the 5′ splice site and an 11-nt C-rich motif at the branch point site in humans [6]. The lariat further undergoes 3′-end trimming to form circular sisRNA. The sequence of this lariat-derived circRNA corresponds to a lariat without a tail but that has kept the 2′–5′ bond [6,23,25] (Figure 1).

## 3. What Can We Expect from Tools Dedicated to the Detection of circRNA?

Novel circRNAs are usually discovered using sequencing datasets generated from total RNA with depleted ribosomal RNA (rRNA) sequences. Reads spanning the circular junction are aligned to the reference genome sequence in two segments mapping in reverse order. The most frequent method of characterizing circRNA is using an available computational tool (reviewed in [26,27]). These detection tools first identify reads spanning the circular junction and subsequently use different methods to improve the reliability of the list of newly described circRNAs.

**Figure 2:**
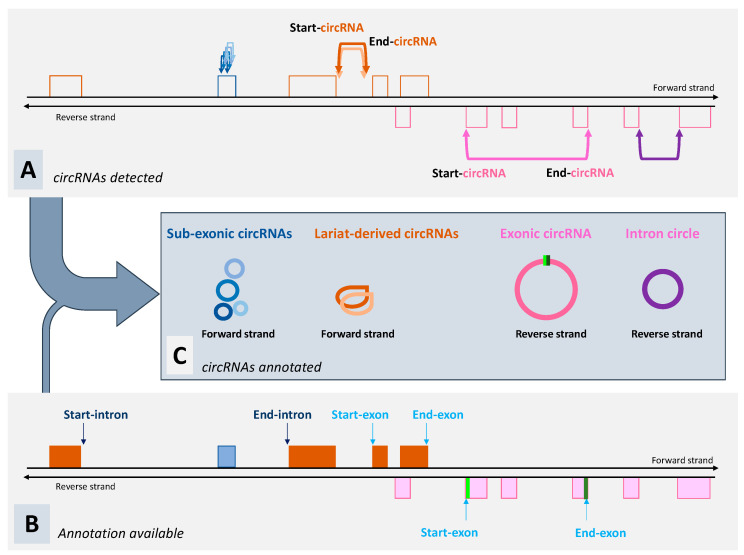
Detection and annotation of circRNAs. (**A**) In this region, several circRNAs (4× blue, 2× brown, 1× pink, and 1× purple) are detected. (**B**) Three genes are described in this region: two on the forward strand (brown gene and blue gene) and one on the reverse strand (pink gene). (**C**) Annotation of circRNAs. The circRNA indicated in pink can be annotated as exonic circRNA (canonical circRNA). In the circular junction sequenced, an exonic downstream donor 3′ splice site (indicated in green in (**B**)) is covalently joined to an exonic upstream 5′ splice site (indicated in dark-green in (**B**)). The parent gene of this exonic circRNA is the pink gene. For the two circRNAs indicated in brown, they are mapped on the forward strand inside intronic sequences of the brown gene located on the same strand. As the position ‘Start-circRNA’ (see (**A**)) is identical to the position ‘Start-intron’ (see (**B**)) and the distance between the position ‘End-circRNA’(see (**A**)) and the position ‘End-intron’ (see (**B**)) is less than 60 nt, we can annotate these two circRNAs as lariat-derived circRNAs from the brown gene. The circRNA indicated in purple is mapped on the reverse strand inside an intron of the pink gene located on the same strand. We can note identical genomic coordinates for ‘Start-circRNA’ and ‘Start-intron’ and for ‘End-circRNA’ and ‘End-intron’. Thus, we can annotate this circRNA as an intron circle produced by the pink gene. When we examine the characteristics of the four circRNAs indicated in blue, we observe that they are compatible with a set of sub-exonic circRNAs produced by the blue gene, which is a non-coding gene. Indeed, their genomic coordinates are located inside the single exon of this gene and the transcription strand is identical.

A circRNA is described by only the two points involved in the circular junction (the genomic boundaries of the circularized transcript: two genomic coordinates) and the strand. Most available computational tools provide a list of circRNAs, including their own analysis of ‘genomic origin’. Nonetheless, we observed that some authors prefer to perform this analysis themselves, especially in non-model species. There are two possible strategies to analyze a list of circRNAs. We call the process that only uses mapping information “classification”; this is based on the location of each of its boundaries on the reference genome and the respective genome annotation at this location. With a classification approach, the list of circRNAs can be divided into several groups according to their overlap with known genetic components such as exons, intron, intergenic, 5′-UTR, 3′-UTR, etc. [7,28]. We prefer another method that we call “annotation”, which is based on a model that explains how a circRNA is generated. For example, the usual method applied to annotate a circRNA as exonic circRNA consists of identifying the two exons whose two borders are joined and are included in reads containing the circular junction (pink-circRNA, Figure 2). For exonic circRNA, the identification of both the exons involved in the back splicing allows for the connection of the circRNA to other linear transcripts containing these exons and finally, the identification of their common parent gene. We assume that it is useful to identify the gene that is likely to generate the circRNA under consideration along with the previously described linear transcripts, namely the parent gene. We note that the annotation approach is often used when authors want to focus their analyses on exonic circRNAs and their parent gene [21,29,30]. We would like to emphasize that considering circRNA as exonic depends on the knowledge of the genome and available annotation. The best described example is circRNA ciRS-7/CDR1as [31]. When this circRNA was characterized, only the protein-coding gene *CDR1* was described at this locus [32], but subsequent studies have shown that the parent gene is a long non-coding RNA located on the opposite strand to *CDR1* [33].

It is possible to produce excellent analyses on circRNAs with a classification approach [7,28], but to identify circRNAs deriving from intron lariat, the annotation approach seems essential to us. Zhang et al. [6] proposed key criteria to identify intron lariat: the first boundary of the circular junction must coincide with the beginning of the intron and the second boundary must be compatible with a circularization event limited by the branch point (brown circRNAs, Figure 2). Zhang et al. [6] proposed to use the term ‘circular intronic RNA’, but we noticed that many studies provide lists of ‘intronic circRNAs’ or ‘circRNAs (deriving) from introns’. Just because the two boundaries identified in the circular junction sequence are mapped in intronic sequences does not necessarily imply a lariat-derived intronic circRNA. We reviewed several published lists in which we expected to find lariat-derived intronic circRNAs and were disappointed, except for a very recent article by Das et al. published in 2020 [24]. In the literature, we can easily find articles that compare computational tools dedicated to circRNA identification [26,27] and we note that they correctly report the consideration of circRNAs when originating from back splicing. In contrast, it is not easy to find the appropriate information for identification of the circRNAs that originate from intron lariats. For example, many authors used a computational strategy to detect circRNAs, including a filter on the canonical splicing motifs. In our opinion, with this filter, it is highly unlikely that a lariat-derived intronic circRNA will be found among the circRNAs detected, even though they may be classified by authors as ‘intronic’. We greatly appreciated to note [24] that CIRCexplorer2 [34] considers two models of circRNA (exonic and lariat-derived intronic). In addition to tools developed to detect “all circRNAs”, we would like to mention a tool designed to work on intronic reads [35] and the development of a dedicated approach to characterize intron-derived circRNAs [20,21]. In 2013, Zhang et al. [6] developed circFinder specifically to study circRNAs derived from introns.

## 4. Review of Reported Lariat-Derived circRNA to Underline Features Related to Their Genesis

Zhang et al. (2013) [6] are considered as the first to report circRNAs derived from lariat introns. In the same year, Jeck et al. [8] described similar circular transcripts but only in datasets obtained after ribo-depletion and linear RNA degradation by RNase-R exonuclease, so they preferred not to validate them (see Section 8). However, already in 2012, Gardner et al. [17] underlined the existence of these intronic transcripts in a dataset obtained after ribo-depletion, but they preferred to connect their observations to sisRNA. Experts in the sisRNAs domain mention that these stable intronic transcripts might be linear [18,19] or circular. Even today, the circular nature of these transcripts has still not been systematically investigated [36] and we emphasize that in the absence of proof, the respective ones are automatically considered to be linear.

To the best of our knowledge, only five studies provide lists of lariat-derived intronic circRNAs. (1) Zhang et al. [6] proposed a list of circRNAs after investigating RNA-seq data obtained after the depletion of both ribosomal sequences and poly-adenylated transcripts. (2) Talhouarne and Gall proposed lists of intron-derived circRNAs, particularly from mouse and human red blood cells [23]. (3) CircRNAs deriving from intron lariats were identified by our group when analyzing datasets of porcine pubertal testes [20,21]. (4) A recent study of neuronal cells (neuronal projections and synapses) in mice identified 278 introns as able to produce sisRNA, and reads containing circular junctions were detected in 14 of them [22]. (5) Das et al. [24] identified circRNAs in mouse pancreatic β-cells and highlighted several lariat-derived circRNAs originating from *Ins2*.

The tricky part of identifying lariat-derived circRNAs is checking that the second boundary of lariat-derived circRNAs is compatible with a circularization event limited by the branch point [6]. Thanks to studies on human branch points, we know a distance of 9–400 bp between the branch point and the 3′ end of the intron is possible [37], even though 90% of branch points occur within 19 to 37 nucleotides upstream of the 3′ splice site [16]. After examining published lariat-derived circRNAs [20,21,23,24], we have no objection considering a distance between the branch point and the 3′ end of the intron ranging from 12 to 60 nt for lariat-derived circRNAs. Moreover, when examining the sequence of reads spanning the circular junction of a lariat derived intronic circRNA, heterogeneity can sometimes be observed, as if several branch points are possible [20,21,24]. Several studies showed that lariat-derived circRNAs that originated from introns did not use an adenosine at the branch point [6,20,21,22,23,24], but sometimes an ‘A’ can be observed. [20,24]. Why are these intron sequence transcripts not removed by a classical intron lariat branched by an ‘A’? More generally, in studies of the sequences involved in the circular junction and of the sequences located downstream of the branch point of introns concerned by lariat-derived circRNA [6,22,25], it would be interesting to compare these sequences with the sequence of introns not concerned by lariat-derived circRNAs (60,000 have already been studied in humans [16]).

The most recent reports suggest that a mammalian genome could produce less than 200 different lariat-derived intronic circRNAs [6,21,22,23]. Published lariat-derived intronic circRNAs seem small and although these observations depend on the authors’ choices concerning the initial analyses, circRNAs derived from introns larger than 1 kb have rarely been characterized [6,20,21,23,24]. We know that very large introns can be spliced in several successive steps based on the formation of several successive intron lariats [38]. Even though the existence of internal branch points could explain circRNAs mapped in large introns, we think that it would be premature to consider that all circRNAs mapped in large introns are lariat-derived circRNAs. Indeed, there are still too many unresolved issues: What defines an internal branch point? What defines a large intron?

## 5. Why Have Lariat-Derived Intronic circRNAs Not Been Better Investigated?

Even though intronic circRNAs deriving from lariats were first described in 2013 [6], only a few articles reported the characterization of this type of circRNA [6,20,21], and now, we will try to explain why. The first reason is the computational tools used that may filter for splicing signal motifs, as explained above. The second possible reason is the presence of a 2′–5′ phosphodiester bond (not a 3′–5′) at the circular junction that may have consequences for the 3D structure of the circular molecule and for its accessibility by reverse transcriptase. Only a few articles report amplification by RT-PCR of the region containing the circular junction of intron lariat-derived circRNA [21,23,24] and in a previous study, we underlined the difficulty in finding the best experimental conditions to obtain an amplicon [21]. We underline that the test of resistance to RNase-R treatment of RNAs [11,12,14], usually proposed to confirm the circular structure, does not appear to be appropriate for these circRNAs (see Section 8).

Very frequently, reads spanning the circular junction region from intron lariat-derived circRNA are under-represented among the reads of the given dataset [21,22,23]. This marked decrease in the base coverage of lariats observed close to the sites of 2′–5′ bonds is compatible with the known inefficient traversal of 2′–5′ junctions by reverse transcriptase [39]. Frequently, an accumulation of reads is observed in the intronic region with indication on an intron lariat-derived circRNA, but surprisingly, reads spanning the circular junction are very rare [21]. In one extreme case, we noted a single read containing the circular junction and several short reads containing only sequences upstream of the presumed circular junction (unpublished data on cow liver). A study by Saini et al. [22] highlighted 278 free intronic transcripts, but we are surprised that reads containing a circular junction were only identified in 14 of them. In contrast to Saini et al. [22], we are not entirely convinced that the preponderant form of sisRNA is linear rather than circular, particularly that of lariat-derived sisRNAs. It is possible that the difficulties encountered by reverse transcriptases beyond the 2′–5′ circular junction are due to the presence of complex secondary structures at this junction. The presence of a 7-nt GU-rich motif upstream of the circular junction [6] is another obstacle to sequencing read-1 of the Pair End (Illumina PE protocols) containing a circular junction derived from a lariat junction [20]. The presence of the 2′–5′ link may have exacerbated consequences for very small circRNAs where we would expect two reads of the pair ends containing the circular junction [20]. Therefore, we assume that they may not have been correctly detected by the bioinformatics tools that usually require coherent information from both mates of a pair [20]. To conclude, we suspect that some of the datasets generated in recent years probably do not reflect the complexity of lariat-derived circular RNAs, probably linked to the preparation of RNA or/and to the preparation of the sequencing library (see Section 8). The use of a reverse transcriptase capable of operating at 65 °C seems to us to be a promising option to study intron lariat-derived circRNA [40].

## 6. Non-Canonical circRNAs Are Even More Poorly Known Than Lariat-Derived circRNAs

The second type of intron-derived circRNAs contain the entire sequence of the intron with a classical 3′–5′ covalent bond, and Taggart et al. [35] suggested naming them “intron circles”. The two boundaries of the circular junction coincide with those of the intron (purple-circRNA, Figure 2), and we observed no under-representation of the circular junction region among the reads. Intron circles have been observed more rarely than lariat-derived intronic circRNAs and appear to be larger [20,23].

In a study using a porcine dataset very rich in circRNAs (SRX5055429 from Testis-31) [20], we observed a large number of reads containing a circular junction and spanning parts of the single exon of *RNase_MRP*. These circRNAs seemed to originate from several circularization events, and this circRNA production cannot be explained by an exonic back splicing. We hypothesized that the transcription product of a gene without intron might undergo a maturation different to that of a classical multi-exonic gene. We extended our search for similar circRNAs to all mono-exonic genes and we noted sets of sub-exonic circRNAs mapped within genes, which were made up of different small circRNAs (maximum 360 nt) containing only a part of a single exon (blue-circRNAs, Figure 2) [20]. Among the 21 genes identified, only two are protein-coding genes but from poorly annotated regions [20]. Thus, we suggest considering in future data analyses that some mono-exonic and non-coding genes are able to produce sub-exonic circRNAs, a new type of non-canonical circRNA. We detected sub-exonic circRNAs originating from ribozyme-RNA, snoRNA, snRNA, misc-RNA, and rRNA genes [20]. The transcription of these genes does not require the splicing step (no intronic sequence to be removed), and these circRNAs could be the first to be produced independently of removing intronic sequences from the pre-messenger. For generating such circRNAs from the single exon of these non-coding genes, we exclude a real splicing event but we do not exclude an action of the splicing machinery, all the more when we know about the role of some of these genes in splicing [41]. So far, each gene characterized as capable of producing sub-exonic circRNAs produces several circRNAs from a single exon and these circRNAs may overlap. Moreover, we never observed these sets of (overlapping) circRNAs from an exon of a multi-exonic gene (observations on SRX5055429 from Testis-31, unpublished in [20]). Even though we know that a few snoRNAs [20,29] are able to produce classical exonic circRNA, we did not detect any mono-exonic gene capable of producing exonic and sub-exonic circRNAs. We would like to underline the fact that also in humans, the production of a set of circRNAs by the mono-exonic gene *RMRP* (orthologous gene of porcine *RNase_MRP*) was highlighted by Liu et al. [42].

In addition, we would like to underline that other types of circular RNAs, generated from tRNAs, rRNA, or C/D box RNAs, are present in Archaea [43]. In animal cells, the splicing of tRNA introns is a critical step in pre-tRNA maturation, even if only a few tRNA genes contain an intron [44,45]. Pre-tRNA is recognizable by the TSEN complex (tRNA splicing endonuclease) and is cleaved by releasing the intron. Lu et al. [44] showed that in animals, a ligase enzyme joins the intron ends together to make a circular RNA, which these authors termed tRNA intronic circular (tricRNA). Even though no tricRNA have been characterized in mammals, the existence of tRNA ligation processing could explain the genesis of intron circles and sub-exonic circRNAs. Indeed, these tRNA ligases could be responsible for the circularization of introns from protein-coding genes into intron circles. However, intron circles could also result from a post-debranching circularization event, as suggested by Taggart et al. [35]. These tRNA ligases could be at the origin of small circRNAs from ribozyme genes [46], which have already been detected among sub-exonic circRNAs [20].

## 7. What Is the Function of Non-Canonical circRNA?

So far, all reported non-canonical circRNAs are derived from intronic sequences (lariat-derived intronic circRNAs or intron circles) or from non-coding genes (sub-exonic circRNAs, tricRNAs). Since we assume that non-canonical circRNAs are derived from non-coding sequences [20], we would expect a function related to the implementation of molecular regulations. So far, we have found no evidence to suggest that sub-exonic circRNAs play a biological role, which does not mean that their biosynthesis has no biological impact. Zhang et al. [6] showed that the transcription of *ANKRD52* is positively regulated by the lariat-derived circRNA produced by one of its introns. These authors suggest that some lariat-derived circRNAs are specifically associated with phosphorylated Pol II and that their depletion leads to a significant reduction in parent gene transcription. Das et al. [24] observed a decrease in the expression of lariat-derived circRNA from an intron of *Ins2* in conditions known to inhibit the mRNA expression from *Ins2*. However, even if these observations are compatible with the hypothesis, they do not prove that this lariat-derived circRNA is able to regulate the transcription of its parent gene. Another observation reported by Talhouarne and Gall [23] is somewhat inconsistent with the hypothesis that these circRNAs regulate the transcription of their own parent gene. Indeed, these authors noted that in the vast majority of genes producing an intron-derived circRNA (lariat-derived or intron circle), most reads appeared to derive from the intron, with very few exonic reads. The same authors also suggest that linear mRNA molecules are lost, while circular RNAs are retained.

In porcine pubertal testes, the lariat-derived intronic circRNA from *ATXN2L* may be between 2 and 40 times more abundant than the linear transcript of this gene depending on the animal [20]. This circRNA may be very small (118 nt) and is the most frequent circRNA in porcine pubertal testis (seven animals). We suggest that for some intron-derived circRNAs, an alternative pathway exists to obtain transcription of the intronic sequence and its circularization independently of the linear transcription of the gene [20]. In the testis of one animal (Testis-31) with high expression of lariat-*ATXN2L*-derived circRNA, expression of *ATXN2L* mRNA was similar (evaluated in mRNA-seq) to that of two animals with a base expression of this lariat-derived circRNA (unpublished in [20]). We observed nothing that supported an impact of this circular transcript on the transcription of the *ATXN2L* gene itself.

Zhang et al. [6] thought that intron-derived circRNAs (lariat-derived or intron circle) resided in the nucleus, while Talhouarne and Gall [25] showed that these transcripts can also be found in the cytoplasm and may therefore, have other functions. It is also possible that intron-derived circRNAs regulate the function of target mRNA at the post-transcriptional level in the cytoplasm. In recent years, the tendency has been to very rapidly generalize from observations of single circRNAs to all circRNAs [13]. Still, even if generic functions are possible, there are certainly many special cases, particularly among non-canonical circRNAs.

Concerning the lariat-*ATXN2L*-derived circRNA in porcine testis, it is questionable whether *ATXN2L* can still be considered the parent gene. In the same line of thought, what would be a good argument to determine the parent gene of a circRNA? A more general question would be what are the limits of a gene? In addition, even more basic, what is a gene?

## 8. How Can We Improve Our Knowledge of Non-Canonical circRNAs?

The sequencing of total RNA with few or no rRNA sequences remains the method of choice and many authors suggest removal of further RNAs prior to sequencing to obtain better sensitivity for circRNAs discovery. In 2013, Jeck et al. [8] were the first to propose treating RNAs with RNase-R to deplete linear transcripts before creating the sequencing library. RNase-R then became a widely used enzymatic tool to work on exonic circRNAs [14], but we suggest to avoid using it for studies on non-canonical circRNAs. Jeck et al. [8] used RNase-R to characterize circRNAs but suggested not retaining the lariat-derived circRNAs detected because they could have been generated by the RNase-R itself. In 2006, the use of RNase-R was proposed to study the branch point of lariat introns [47] and since then, this enzymatic tool has been widely used for this purpose [16,35,47,48]. Talhouarne and Gall [23] used RNase-R combined with transcription inhibition to study lariat-derived circRNAs. An alternative way to improve the sequencing power of non-canonical circRNA is to add a depletion of poly (A+) transcripts, as proposed by Zhang et al. [6].

Non-canonical circRNAs appear to be small, with a large proportion of them comprising less than 400 nucleotides. In a porcine testicular dataset, we observed an average size of 280 nt for lariat-derived circRNAs [20]. However, our experience shows that the identification of very small circRNAs (< 150 nt) is relevant and involves almost exclusively non-canonical circRNAs. Nevertheless, we observed that some groups of datasets contain many more small circRNAs than others, suggesting that protocols to prepare RNA with few or no rRNA and/or protocols for preparing the sequencing library could be important. For example, we suspect that many library preparation protocols for RNA-seq include a size selection step, which is not always explicitly reported. In standard sequencing protocols (Illumina) that have been used since 2012, a fragmentation step is included with the aim of fragmenting long RNAs but also to destroy secondary structures to improve access for the reverse transcriptase. This step consists of a few minutes of high temperature treatment under the buffer conditions proposed by Illumina. For small circRNAs, fragmentation of RNAs (cutting) and denaturation have conflicting interests. Boivin et al. [49] reported that the detection of medium-size non-coding RNAs (20–500 nt) could be unreliable using the standard library preparation for sequencing due to their highly secondary and tertiary structured nature. The use of a reverse transcriptase operating at 65 °C could be a solution for reliable sequencing of these RNAs [40]. We are convinced that there are still many ways to improve the sequencing of non-canonical circRNAs but also of all other RNAs.

The quantification of small circRNAs is difficult by qRT-PCR due to the risk of rolling-circles. New methodologies were developed to quantify circRNAs directly on RNAs, without the use of a reverse transcription [50,51]. These two methodologies are based on knowledge about existing circular junctions. Nevertheless, the heterogeneity at the point of circularization and the nature of sequences inadequate for probe design can impede setting up respective experiments, e.g., for SplintQuant (own results, not published).

Numerous tools for circRNA detection are available [26,27]. Whatever the qualities and characteristics of these tools, the quality of a circRNA screening will also depend on the user. It seems important to us to avoid applying these bioinformatics tools as a simple black box. Users should try to understand the filtering options included in the pipeline. For example, the inclusion of a filter on canonical splicing motifs makes it highly unlikely to detect lariat-derived intronic circRNAs or more generally, non-canonical circRNAs. To access non-canonical circRNAs, we suggest a bioinformatic strategy removing all reads assigned to exonic circRNAs. To do this, the first method is to identify all possible exonic circRNAs by an annotation process [20] and the second would be to discard all exonic circRNAs using a filter based on canonical splicing patterns and to make a selection based on the genomic size of circRNAs (< 800 bp). No methodology is perfect: the identification of exonic circRNAs through an annotation process depends on the available annotation and a small share of identification errors using a filter based on canonical splicing models is expected. We would like to emphasize that when we propose to search for non-canonical circRNAs preferentially among non-exonic circRNAs, this does not mean that all circRNAs, that we are not able to identify as exonic circRNAs, should be considered as non-canonical circRNAs. At the end of the annotation process of a list of circRNAs and even after the removing of sporadic circularization events (filtering step that we encourage), there are still some circRNAs without annotation. We can only suggest performing additional analyses (RT-PCR, examination of sequences in reads containing circular junctions, review of annotations available at loci concerned…) before considering them as new non-canonical circRNA.

## 9. Conclusions

Even though we suspect that most of the datasets generated by sequencing in recent years probably do not allow for full appreciation of the complexity of non-canonical circRNAs, we believe that the sequencing of total RNA with few or no rRNA sequences remains the best method for the study of circRNAs. We show that there are still several possible ways to improve the sequencing and consequently, our knowledge of non-canonical circRNA. To study non-canonical circular RNAs, it is sometimes preferable to deviate from conventional approaches, and for example, we can suggest moving aside the thick curtain formed by exonic circRNAs to access those non-canonical circRNAs. We hope this review will inspire others to continue studies of these poorly understood classes of circRNAs.
